# Researchers aren't the story: why we should cite theories and findings, not people

**DOI:** 10.3389/fpsyg.2025.1663529

**Published:** 2025-09-05

**Authors:** Robert Körner

**Affiliations:** Department of Psychology, University of Bamberg, Bamberg, Germany

**Keywords:** indirect citation, citation behavior, authorship, illusion of control, intentional stance

Authors, especially those of review papers or book chapters, often explicitly mention the name of a specific researcher or research group when describing an experimental procedure that led to the detection of a phenomenon, or they highlight the big name behind a theory. Here, I argue that such practices, despite being popular, are subject to a variety of illusions and biases, and that referring to researchers indirectly (in parentheses, at the end of sentences) would be more advisable.

## Simultaneous discoveries

Letterpress printing was invented by Johannes Gutenberg in 1450—a fact many pupils learn in school. However, it's less well known that, 400 years earlier in China, another one (Bi Cheng) invented another form of letterpress printing (Needham, 1974). Despite using a different technique, Cheng's work was recognized by Europeans in the fourteenth century, and craftsman in Italy and the Netherlands worked on similar printing methods. So, would there be no letterpress without Gutenberg? Similarly, Thomas Alva Edison is commonly viewed as the inventor of the light bulb. However, several other people worked on prototypes or earlier versions that brought wires to light (Friedel and Israel, 2010). So, would we sit in the dark at night today if there had been no Edison?

These two examples briefly illustrate that important historical discoveries cannot be easily attributed to a single person. The invention of new technologies and the progression of knowledge would likely have occurred even without the people we most strongly associate with them. It could be argued that historical narratives often favor clear, single names (like Gutenberg, Edison) because they are easier to teach and remember (Basalla, 2010; Brooks, 2024). However, innovation is typically cumulative and collaborative—many people contribute in overlapping ways, but fame often comes down to the person who commercialized or popularized the invention most successfully (Bazerman, 1999).

This also applies to several psychological theories and phenomena that were discovered—or at least described—around the same time by independent researchers or labs, but only gained widespread recognition through later researchers. For examples across social, personality, developmental, and cognitive psychology, see Table 1. Moreover, psychological research often suffers from the jangle fallacy, meaning that two constructs are identical but are labeled differently (Block, 1995; Lawson and Robins, 2021). Researchers may gain recognition for introducing a “new” concept, even though it might be the same as an existing one under a different name (see Altgassen et al., 2024; Ponnock et al., 2020). Focusing on the underlying construct or theory and citing that theory rather than individual researchers who use different labels can help avoid such jangle fallacies.

**Table 1:** Psychological theories and phenomena discovered by independent researchers.

**Theory or phenomenon**	**Usually cited source**	**Other important work**
Attachment theory	Bowlby (1969)	The importance of social ties for emotional development was demonstrated a decade earlier by studies on primates (Harlow, 1958)
Basic emotions	Ekman (1992)	A “basic emotion” approach, proposing nine basic affects (interest, enjoyment, surprise, distress, fear, anger, shame, dissmell, and disgust), had already been introduced (Tomkins, 1962)
Fundamental attribution error	Ross (1977)	Ten years earlier, it was shown that participants could not refrain from attributing dispositions rather than situational causes. Participants read essays either supporting or opposing Castro and rated the authors' true attitudes. Even when told that the positions were assigned randomly (by a coin toss), participants judged pro-Castro writers as genuinely more pro-Castro—they did not interpret the speakers as merely performing a task (Jones and Harris, 1967)
Theory of mind	Premack and Woodruff (1978); (Wimmer and Perner (1983)	Although both sources are heavily cited, they underscore the importance of independently detecting a phenomenon (e.g., understanding others' mental states)
Facial feedback hypothesis	Strack et al. (1988)	Others had manipulated facial expressions even earlier and reported downstream consequences (e.g., Laird, 1974; Lanzetta et al., 1976)
Power posing	Carney et al. (2010)	Posture manipulations were used 30 years earlier to examine their effects on thoughts, feelings, and behavior (Riskind and Gotay, 1982)

## Psychological biases in understanding scientific progress

Simultaneous discoveries support the validity, robustness, and generalizability of phenomena, but they also show that often no single person or research group is completely responsible for their exploration (Merton, 1961). Rather, they suggest that some phenomena are “in the air” (e.g., due to newly available methods, pressing societal issues), and would be discovered sooner or later (Kelly, 2010). Thus, attributing a unique contribution to any particular individual or research lab is limited by these considerations. This may even lead to the strong conclusion that attributing discoveries to a single researcher may be misleading. However, I do not intend to diminish the contributions of those who help develop or detect new psychological phenomena or technologies. In fact, we are all subject to the *illusion of control* (Langer, 1975; Presson and Benassi, 1996), that is, we believe we control things we actually cannot control because they are determined by external forces. In other words, no single person fully controls the emergence of new theories or phenomena, because scientific, societal, historical, and cultural factors often have a stronger influence than any one individual. Typically, chance plays a stronger role than we assume (Frank, 2016; Pluchino et al., 2018). Similarly, the *intentional stance* (Dennett, 1989) suggests that we interpret people's actions as purposeful. Again, we tend to be biased in our perceptions: We overestimate internal factors, such as the intention to develop a new theory, and underestimate external factors. In social psychology, this is similar to the fundamental attribution error (or correspondence bias, Gawronski, 2004).

Moreover, when someone gains attention for a new theory or phenomenon, it can spread widely: The more often people read about the new theory, the more they may like it (mere-exposure effect, Zajonc, 1968). Thus, people (or even universities or countries) who are good at marketing their research may also be rewarded by people liking their research. For example, confident individuals and people in countries with a more individualistic orientation may be especially skilled at promoting their ideas (Avia et al., 1998; Hofstede, 1980) and, in turn, be more likely to receive credit. This can lead others to adopt the new theory in their own work, eventually making the theory more visible and popular because people use it and talk about it (social contagion, Levy and Nail, 1993). Over time, this creates a self-reinforcing cycle in which the theory becomes increasingly successful. Psychological research supports the idea that there is truth in the saying, “To him that hath shall be given” (Matthew effect, Merton, 1968). For example, theories with many citations often continue to get even more attention and citations, because people think that this is a key work (Beel and Gipp, 2009; Perc, 2014). Therefore, it is advisable to evaluate the content of a theory rather than relying solely on its citation count.

## Conclusions for citing behavior

Now that I have briefly argued that theories and phenomena are often not attributable to a single person or research group—and that several psychological biases lead to some researchers being placed on a pedestal while others do not receive similar recognition—what does this mean for citation practices? When a researcher's name is used as the subject of a sentence, it reinforces the illusion of sole discovery or intent (“this researcher is solely responsible for intentionally detecting this phenomenon”), which boosts the popularity of the research (social contagion processes and “success breeds success”) more than when the focus is on the theory or phenomenon itself. This practice may even be irrational, especially when the “founders” of key theories are no longer alive or active—meaning that citing their work is unlikely to lead to more favorable peer reviews or publication decisions. By using indirect citations (referring to researchers in parentheses), we still acknowledge their scientific contributions, but we shift the focus away from the individual. Instead, we emphasize the theory, phenomena, or finding (e.g., Bem, 1995). In addition, indirect citations often improve flow and readability. For example, instead of writing, “Bem (1995) suggests using indirect citations to focus on findings,” it is recommended to write “Indirect citations can shift the focus to findings (Bem, 1995).”

Further, if there are multiple people associated with a theory or phenomenon, their names can be placed in parentheses at the end of the sentence (“e.g.,”) to denote that the list may not be exhaustive. Similarly, if there are multiple studies that demonstrate a finding, citing a meta-analytic review in parentheses at the end of the sentence would be advisable. These practices improve readability compared to listing names at the beginning of a sentence. Such considerations—along with the fact that placing names at the end of sentences shifts the focus from the people to the theory or finding—are especially important for students who try to memorize theories and names for exams. In fact, it may even be advisable to avoid testing names in exams, in order to reduce biases related to sole discovery or the Matthew effect, as outlined above. Indirect citations are also important for other audiences who might “get lost in the weeds,” such as laypeople, politicians, journalists, or pediatricians. Overall, using indirect citations may help reduce psychological biases and illusions that contribute to inequality in the recognition of scientific contributions.
